# Dysregulation of RNA Splicing in Tauopathies

**DOI:** 10.1016/j.celrep.2019.11.093

**Published:** 2019-12-24

**Authors:** Daniel J. Apicco, Cheng Zhang, Brandon Maziuk, Lulu Jiang, Heather I. Ballance, Samantha Boudeau, Choong Ung, Hu Li, Benjamin Wolozin

**Affiliations:** 1 Boston University School of Medicine, Department of Pharmacology and Experimental Therapeutics, Boston, MA, USA; 2 Mayo Clinic, Rochester, MN, USA; 3 Boston University School of Medicine, Department of Neurology, Boston, MA, USA; 4 These authors contributed equally; 5 Lead Contact

## Abstract

Pathological aggregation of RNA binding proteins (RBPs) is associated with dysregulation of RNA splicing in PS19 P301S tau transgenic mice and in Alzheimer’s disease brain tissues. The dysregulated splicing particularly affects genes involved in synaptic transmission. The effects of neuroprotective TIA1 reduction on PS19 mice are also examined. TIA1 reduction reduces disease-linked alternative splicing events for the major synaptic mRNA transcripts examined, suggesting that normalization of RBP functions is associated with the neuroprotection. Use of the NetDecoder informatics algorithm identifies key upstream biological targets, including MYC and EGFR, underlying the transcriptional and splicing changes in the protected compared to tauopathy mice. Pharmacological inhibition of MYC and EGFR activity in neuronal cultures tau recapitulates the neuroprotective effects of TIA1 reduction. These results demonstrate that dysfunction of RBPs and RNA splicing processes are major elements of the pathophysiology of tauopathies, as well as potential therapeutic targets for tauopathies.

## In Brief

RNA binding proteins are emerging as drivers of neurodegeneration. Apicco et al. show a dysregulation of RNA splicing in mouse and human tauopathies, with partial correction by reducing TIA1. A systems analysis suggests novel approaches for the therapy of tauopathy.

## Graphical Abstract

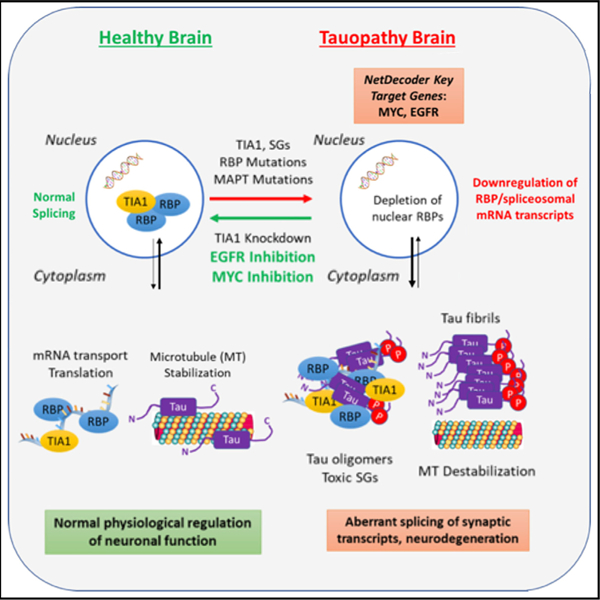

## INTRODUCTION

Microtubule associated protein tau (MAPT or tau) normally binds microtubules to stabilize the axonal cytoskeleton. In a collection of neurological disorders termed tauopathies, tau becomes mislocalized to the somatodendritic compartment where it misfolds and aggregates into insoluble inclusions termed neurofibrillary tangles (NFTs) (Zempel and Mandelkow, 2014). Despite being the defining pathological hallmark of tauopathy, little is understood about the potential pathological functions and mechanisms of mis-sorted tau. Our group recently discovered that somatodendritic tau exerts an important biological function in regulating the translational stress response and the biology of RNA binding proteins (RBPs). This tau-mediated stress response is associated with a shift in protein synthesis and an increase in the formation of stress granules (SGs) that can lead to sequestration of RBPs in the cytoplasm (Vanderweyde et al., 2016). We also demonstrated that in pathological murine or human tauopathy, somatodendritic tau co-localizes with TIA1, an RBP with an intrinsically disordered region (IDR, also referred to as a low-complexity or prion-like domain) that functions as a core nucleating SG protein, which stimulates the regulated aggregation of additional RBPs in the cytoplasm (Anderson and Kedersha, 2008; Vanderweyde et al., 2012, 2016). The role of somatodendritic tau in the biology of RBPs also suggested that RBPs might reciprocally regulate the pathophysiology of tau. Pursuing this hypothesis, we recently demonstrated that reduction of TIA1 in the PS19 P301S tau mouse model results in a delay of neurodegeneration, protection against behavioral deficits and a prolongation of lifespan (Apicco et al., 2018). Further, these mice exhibited reduced nuclear-to-cytoplasmic translocation of TIA1, reduced formation of cytoplasmic RNA granules containing markers of SGs, and increased stabilization of microtubules (Apicco et al., 2018). The disease modifying effects of TIA1 reduction, the colocalization of TIA1 with somatodendritic tau, and the effects of somatodendritic tau on SG accumulation raise the possibility that dysfunction of RNA metabolism might be an important contributor to the pathophysiology of tauopathy.

Here, we assessed whether tauopathies are associated with deficits in RNA metabolism. Our results demonstrate a significant dysfunction of RNA splicing in both the PS19 tau mouse model and in the Alzheimer disease (AD) brain. Importantly, we also show that reduced expression of TIA1 in PS19 mice normalizes some of the disease-related changes in RNA splicing. We then used an unbiased systems biology approach to identify upstream regulators that might contribute to the transcriptional and splice variant changes in the neuroprotected PS19 *Tia1*^+/−^ mice compared to the PS19 *Tia1*^+/+^ tauopathy mice. We identify several targets, including MYC and the EGF receptor (EGFR), that drive the pathological changes in both PS19 mice and primary neuronal cultures. This work suggests that dysregulated splicing is an important pathophysiological mechanism in tauopathies and identifies therapeutic approaches for disease modification.

## RESULTS

### RNA Metabolic Processes Are Dysregulated in PS19 Tauopathy Mice

In order to determine how the RNA transcriptome is altered in PS19 tauopathy mice, we isolated total RNA extracts from the cortex of 9-month-old wild-type (WT), PS19 (P301S *Tia1*^+/+^) and P301S *Tia1*^+/−^ mice (refer to the STAR Methods for a detailed description of the breeding scheme). We chose to analyze the transcriptome at the 9-month time point because this corresponds to an age where PS19 mice are severely affected by tauopathy, while P301S *Tia1*^+/−^ mice are largely protected from the neurological and motor symptoms of disease (Apicco et al., 2018); non-transgenic tau mice exhibit no symptoms of disease or abnormal aging at 9 months regardless of *Tia1* genotype.

**Figure 1.**
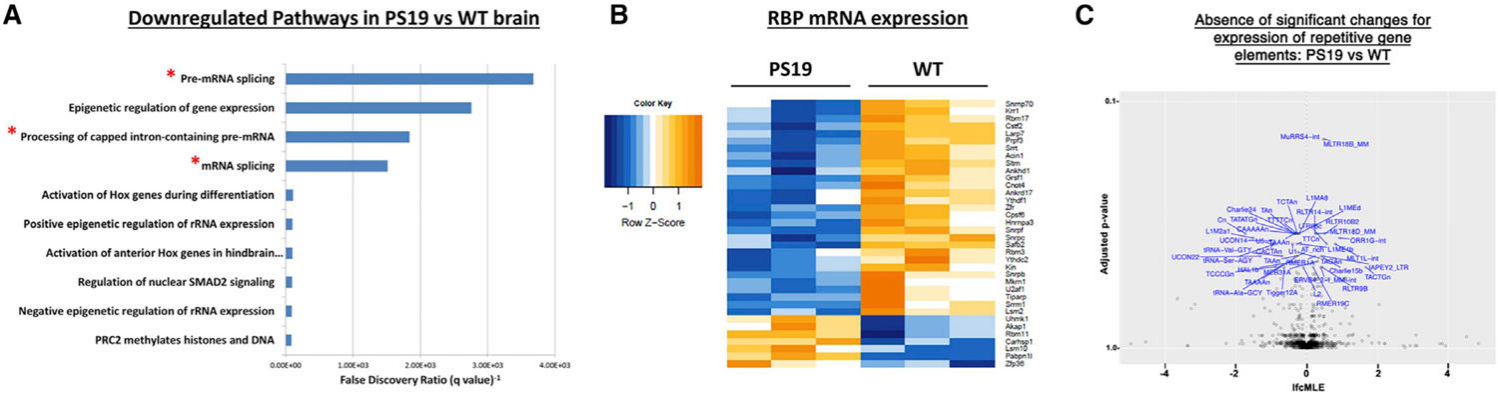
Transcripts Encoding Spliceosomal Complex and mRNA Binding Proteins Are Downregulated in PS19 Brain (A) Top 10 most statistically significant BioCarta reactome pathways enriched in the gene list of mRNA transcripts downregulated (Fisher’s adjusted p value < 0.05) in 9-month PS19 compared to WT cortex tissue, as determined by GSEA. Red asterisks denote annotation terms related to mRNA splicing or mRNA processing. (B) Heatmap of mRNA expression levels of transcripts encoding RNA binding proteins (RBPs) in the RNA-seq of PS19 compared to WT cortex. RBPs were defined by inclusion of the GO molecular function annotation ‘‘RNA-binding.’’ Note that 30 of the 37 RBPs identified were decreased in PS19 compared to the WT samples. Blue to red color scale denotes negative to positive *Z* scores (color key, left). (C) Volcano plot for repetitive elements in 6 months in PS19 *Tia1*^+/+^ compared to WT mouse cortex. p values (y axis) are plotted against the log2-transformed fold change (x axis) in the level of each repetitive element transcript. No elements showed statistically significant changes.

We first used next-generation RNA sequencing (RNA-seq) to analyze the differential expression of mRNA levels in PS19 compared to WT mice. 339 genes were discovered to be significantly up- or downregulated (FDR < 0.05) in the PS19 cortex (Table S1). Gene set enrichment analysis (GSEA) was performed using the whole transcriptome ranked by fold changes against the gene sets described in Enrichment Map (Subramanian et al., 2005; Merico et al., 2010), which covers pathways and Gene Ontology (GO) annotations of the mouse genome. 43 pathways showed significant negative enrichment (downregulation) (Table S2), including pre-mRNA splicing (false discovery rate [FDR] = 2.7E–4), processing of capped intron-containing pre-mRNA (FDR = 5.4E–4), and mRNA-splicing (FDR = 6.6E–4). Further, the topmost significant downregulated GO biological process and cellular component terms were RNA splicing (FDR = 1.10E–2) and spliceosomal complex (FDR = 1.1E–3), respectively (Table S2). In contrast, no pathway gene sets were positively enriched with statistical significance, suggesting that there is not a strong biological trend in the genes that become upregulated in tauopathy. Analysis of the downregulated pathways in the PS19 compared to non-transgenic mice suggests that abnormal processing of mRNA, especially RNA splicing, is one of the predominant features of tauopathy (Figure 1A; Table S2).

We next examined whether the downregulation of pathways related to RNA metabolism reflected an underlying reduction in the expression level of mRNA-encoding spliceosomal proteins and RBPs. Expression-level analysis was performed for mRNA data that were encompassed by the GO molecular function annotation ‘‘RNA-binding’’ (Figure 1B). Of the 37 RBPs transcripts identified, 30 were decreased in the PS19 compared to the WT samples (Figure 1B), including Snrnp70, Rbm 11/17, Hnrnpa3, Snrp b/c/f, and U2af1, which indicates a strong response of RBP biology in the PS19 mouse brain. About 25% of these proteins (10/37) participate in spliceosomal function, including Snrp 70, b/c/f, U2af1, LSM2, 10, Safb2, Prpf3, and Srrm1.

Since previous results in *Drosophila melanogaster* demonstrated striking re-expression of transposable elements in response to the TDP-43-mediated degenerative process (Li et al., 2013), we also examined the expression of transposons in the PS19 model. Transcripts mapping to multiple different genetic locations were identified and quantified in our RNA-seq data. No transposable elements exhibited significant differences in expression in PS19 compared to WT mice (Figure 1C). Combined, these data suggest that the genetic dysregulation caused by tauopathy is selective to particular pathways and that a reduction of RNA-splicing processes is particularly notable.

### Alternative Splicing of Genes Encoding Synaptic Proteins Is Disrupted in PS19 Mice

**Figure 2.**
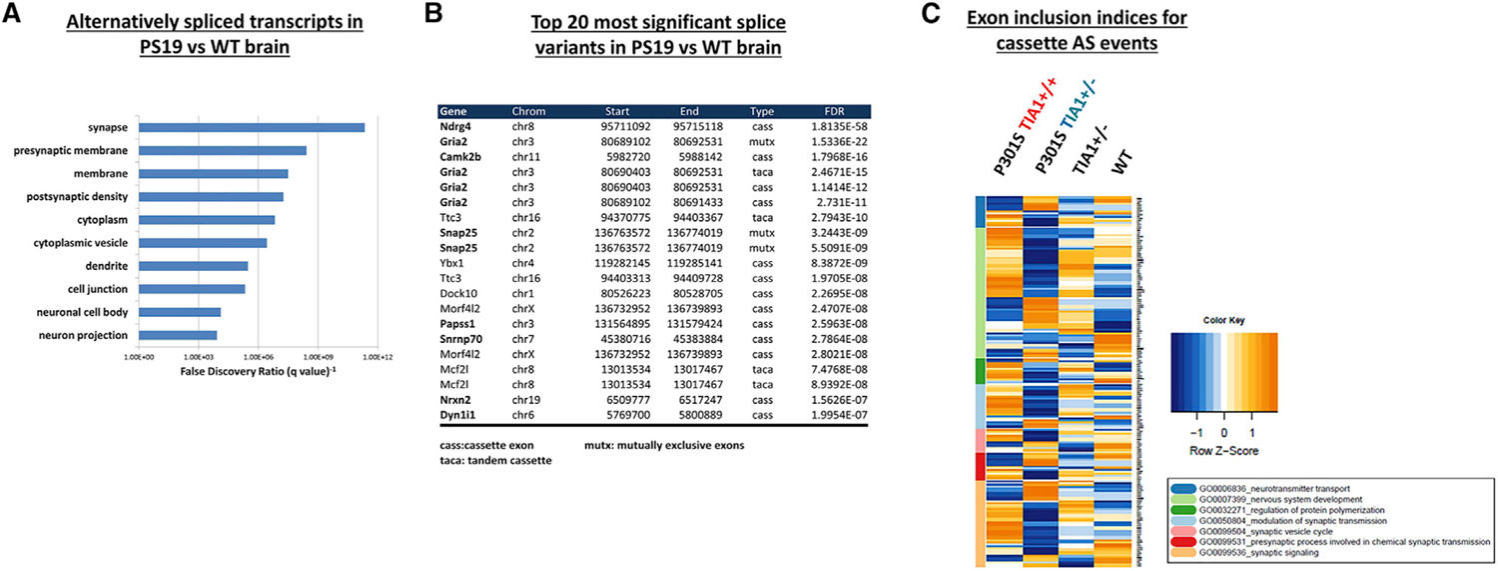
mRNA Transcripts Encoding Synaptic Proteins Are Alternatively Spliced in PS19 Brain (A) For PS19 versus WT frontal cortex, top 10 most statistically significant Gene Ontology (GO) biological process annotation terms enriched (FDR < 0.05) in the gene list of mRNA transcripts exhibiting significant changes in splicing, as determined by OLego and Quantas splice variant analysis. (B) Top 20 most statistically significant individual splice variants in PS19 compared to WT cortex. Note that 11 of the top 20 alternatively spliced transcripts encode proteins with known functional roles at the synapse (bold). Note that some genes appear on the list multiple times because a change in mutually exclusive exons will also be recorded as a significant cassette (exon skipping) event, etc. Please refer to Tables S3 and S6 for the complete list of alternative splicing results. (C) Hierarchically clustered heatmap of exon inclusion indices for alternatively spliced cassette exons in RNA-seq of 9-month PS19 *Tia1*^+/+^, PS19 *Tia1*^+/−^, *Tia1*^+/−^, and WT cortex. Inclusion indices for statistically significant (FDR < 0.1) cassette exon alternative splicing (AS) events were calculated by dividing the number of reads including a given exon where an inclusion/skip occurs by the total number of reads for that transcript. *Z*-transformed inclusion indices were then plotted in a heatmap to visualize changes in exon inclusion between genotypes. Genes were hierarchically clustered according to GO analysis (box inset, below). Note that Tia1 reduction in PS19 mice (P301S tau *Tia1*^+/−^, green) returns the majority of transgenic tau-induced changes in PS19 mice (P301S Tau *Tia1*^+/+^, red) toward WT levels, particularly in the nervous system development (light green), presynaptic process (dark red), and synaptic signaling (orange) GO clusters. Blue to red color scale denotes negative to positive *Z* scores (color key, right).

The observation that RNA-splicing pathways are downregulated in PS19 mice led us to investigate the specific mRNAs that were subject to alternative splicing. Thus, we further analyzed our RNA-seq data using the OLego and Quantas programs to identify significant alternative splicing events in the PS19 compared to WT brain (Wu et al., 2013). OLego detects six different types of alternative splicing events: cassette exons (skipped exon), tandem cassettes (exon duplication), mutually exclusive exons (mutually exclusive inclusion of one of two alternative exons), alternative 5′ splice site selection, alternative 3′ splice site selection, and intron retention. Cassette exons were by far the most frequently observed event in our RNA-seq data, constituting 58.6% of detected events, followed by tandem cassettes (13.9%), intron retention (13.0%), alternative 3′ or 5′ splice site selection (8.6%), and mutually exclusive exons (4.9%). We then analyzed the list of alternatively spliced mRNA transcripts using the DAVID bioinformatics tool (Huang da et al., 2009) in order to describe the biological functions of alternatively spliced genes, and to gain insight into the cellular functions affected by abnormal splicing in tauopathies. Analysis of the list of genes exhibiting statistically significant (FDR-adjusted p values < 0.05) splice variants in PS19 compared to WT brain revealed an enrichment in bioinformatic terms related to synaptic neurotransmission. The top 10 most significant GO biological process annotation terms included synapse (FDR = 6.23E–12), presynaptic membrane (FDR = 2.06E–10), postsynaptic density (FDR = 6.42E–8), and dendrite (FDR = 3.83E–6) (Figure 2A; Table S4). These data suggest that a number of RNA transcripts involved in neurotransmission and synaptic structure/function are subjected to aberrant splicing induced by tauopathy.

**Figure 3.**
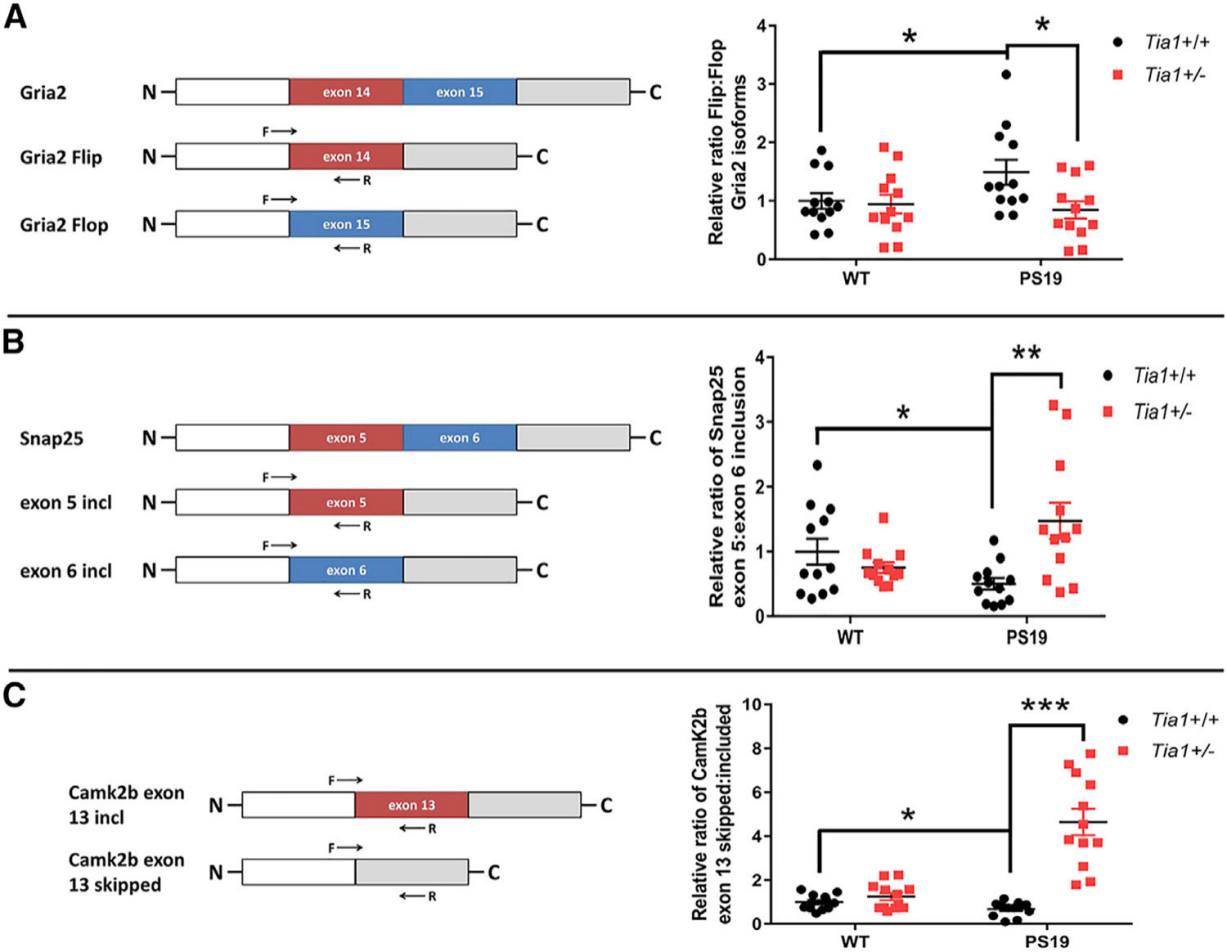
TIA1 Reduction Rescues Dysfunctional Splicing of Synaptic mRNAs in PS19 Brain Relative exon inclusion for statistically significant (FDR < 0.05) alternative splicing events in PS19 (P301S *Tia1*^+/+^) compared to WT cortex was confirmed for Gria2 (A), Snap25 (B), and Camk2b (C) by real-time PCR. Left panels depict exon structure and forward (F) and reverse (R) primer design for each transcript. Ratios of exon inclusion were quantified by calculating the relative expression of each splice isoform compared to a housekeeping transcript (∆DCt Gapdh), normalizing each splice isoform to the total transcript level for each gene, and dividing the relative expression of isoform 1 to isoform 2. All ratios were then normalized to WT ratio = 1; thus, the quantification in the graphs to the right represent relative (and not absolute) changes in the ratio of splice isoforms. *p < 0.05, **p = 0.0025, and ***p < 0.0001 by two-way ANOVA with Tukey’s post hoc comparisons. Error bars denote means ± SEM (n = technical duplicates of 6 mice/group; 3 males and 3 females per genotype).

We next investigated the specific splice isoforms and exons most significantly affected in PS19 mice (Figures 2B and 2C). Figure 2B summarizes the top 20 most significant alternative splicing events detected in PS19 compared to the WT cortex. Notably, 10 of these 20 transcripts encode proteins with known roles at the synapse (Figure 2B, bolded names), including GluR2 (Gria2), CaMK2b, Dlgap2, and Snap25. Further, several of the affected exons have known functional effects on the resulting protein isoforms. Our RNA-seq data show that the ratio of exon 14 (Flip isoform) to exon 15 (Flop isoform) inclusion in Gria2 is elevated almost 2-fold in PS19 compared to the WT cortex (Table S3), producing an increase in Flip GluR2-containing AMPA receptors—a process that causes AMPA receptors to desensitize more slowly, rendering the cell more susceptible to excitotoxicity (Koike et al., 2000; Pei et al., 2009). The RNA-seq data also identified alterations in two other critical synaptic genes with known roles in synaptic transmission, demonstrating that both Snap25 and Camk2b are alternatively spliced in the PS19 cortex (Figure 2B; Table S3). Performing qPCR on RNA extracted from fresh sets of tissue demonstrated the validity of the changes observed in the PS19 samples (Figures 3A–3C). Taken together, the high prevalence in PS19 mice of dysfunctional RNA splicing in genes encoding proteins involved in glutamatergic synaptic transmission suggests that dysregulation of this system could be an important contributor to memory deficits in tauopathies.

### TIA1 Reduction Partially Rescues Dysfunctional Synaptic Splicing in PS19 Mice

Our previous work had shown that reducing TIA1 expression in the PS19 mouse model led to a decreased tau-mediated SG response, reduced tau oligomer accumulation, and increased the lifespan in these mice (Apicco et al., 2018). Given the current identification of a global dysfunction in RNA splicing in PS19 mice and the role of TIA1 in RNA metabolism, we investigated whether TIA1 reduction might also affect alternative splicing.

First, we analyzed whether the 174 genes detected as significant cassette splice variants in our PS19 versus WT mice also exhibited TIA1 binding signatures by comparing our RNA-seq data to previously published TIA1 data from individual nucleotide resolution ultraviolet crosslinking and immunoprecipitation experiments (iCLIP) (Wang et al., 2010). We observed that 49 out of the 174 genes (28.2%, p = 0.019) exhibited TIA1 binding peaks in the iCLIP analysis, suggesting that TIA1 dysfunction partially contributes to the splicing defects in PS19 mice (Table S3, second tab).

We proceeded to compare the relative levels of alternative splice isoforms of mRNA transcripts in P301S *Tia1*^+/−^ mice with those in P301S *Tia1*^+/+^ mice. GO analysis of alternatively spliced transcripts in P301S *Tia1*^+/−^ compared to the P301S *Tia1*^+/+^ cortex revealed that the top-three most significant annotation terms are synapse (FDR = 3.86E–12), postsynaptic density (FDR = 4.33E–12), and presynaptic membrane (FDR = 1.14E–10) (Table S5). While indicating a clear effect on splicing, this result could not distinguish between an increased dysregulation of (i.e., exacerbation) and a decrease in (i.e., rescue) splicing changes relative to WT mice. We therefore examined the inclusion indices for all cassette exon splicing events detected between all four genotypes to determine how TIA1 reduction in PS19 mice affected tau-mediated splicing defects. TIA1 reduction decreased the transgenic tau-induced changes in the exon inclusion index of 195 out of 284 (68.7%) total cassette exons reverting toward WT levels, suggesting an overall net improvement in splicing toward the WT condition (Table S6). Figure 2C summarizes the effect of TIA1 reduction on exon inclusion in WT and PS19 mice in a hierarchical clustered heatmap. Normalization to WT exon inclusion levels was especially prominent for transcripts encoding proteins with GO annotations related to nervous system development and synaptic transmission (Figure 2C), suggesting that splicing-mediated alterations in synaptic transmission play a role in the pathophysiology of tauopathy. Interestingly, TIA1 reduction in non-tau transgenic mice altered exon inclusion in the direction of PS19 tauopathy mice for some transcripts despite there being no overt change in neurodegenerative phenotype (Figure 2C). This result suggests that TIA1 acts to shift the splicing of some transcripts relevant to neurodegeneration in a manner that is independent of the presence of overt neurodegeneration.

We next investigated whether TIA1 reduction corrected the splicing alterations in the specific excitatory synaptic transcripts most significantly changed in PS19 compared to the non-transgenic cortex (Figure 2B), including Gria2, Snap25, and Camk2b. We designed primer pairs that spanned exon junctions of transcripts including or excluding the affected exons, as well as primers to amplify total transcript levels (spanning constitutive exons) for normalization of each splice isoform (Figures 3A–3C). Real-time PCR analysis of cortex tissues collected from new 6-month-old mice (n = 6/genotype; 3 males and 3 females per group) confirmed that the ratios of exon inclusion were statistically different in PS19 compared to the WT cortex for all three transcripts (Figures 3A–3C); these results are similar to those reported recently by Hsieh et al. (2019). More importantly, TIA1 reduction restored the ratio of exon inclusion versus exclusion toward the levels detected in WT mice for all three transcripts (Figures 3A–3C). While this result does not demonstrate a direct role for TIA1 in mediating alternative splicing of Gria2, Snap25, or Camk2b, the results indicate that normalization of splicing represents a clear component of the neuroprotection provided by TIA1 reduction in tauopathy.

We also compared transcriptional profiles of P301S *Tia1*^+/+^ and P301S *Tia1*^+/−^ mice (Figures S1 and S2; Table S1). The results showed a large number of genes whose transcriptional profiles were changed by *Tia1* haplo-insufficiency (Table S6b). However, the FDR-adjusted p values for the top 10 most significant GO annotation terms revealed did not produce as strong enrichment values as observed for the splicing changes (Table S7).

### TIA1 Heterozygosity Regulates Expression of Sexually Dimorphic Genes

The RNA-seq data also revealed a surprising role for TIA1 in regulating sexually dimorphic genes. Analysis of the transcriptomes in WT and *Tia1*^+/−^ mice showed modest changes (<50%) in expression for 928 genes, but dramatic regulation of the long non-coding RNA (lncRNA) Xist, and four genes on the Y chromosome normally silenced by Xist. The top 5 differentially expressed transcripts all encode sexually dimorphic proteins, including Ddx3y (FDR = 6.24E–284), Uty (FDR = 3.62E–212), Xist (FDR = 3.32E–209), Eif2s3y (FDR = 1.09E–169), and Kdm5d (FDR = 2.99E–121) (Table S8). Transcripts expressed on the Y chromosome (Ddx3y, Uty, Eif2sy, and Kdm5d) were significantly reduced in *Tia1*^+/−^ compared to the WT cortex, while levels of transcripts expressed from the X chromosome such as Xist were significantly elevated (Figure S2A). Independent analysis of mRNA transcript levels in new 6-month-old mice (n = 6/group; 3 males and 3 females per genotype) confirmed that *Tia1* heterozygosity significantly increased expression of Xist and reduced expression of Ddx3y, Uty, Eif2s3y, and Kdm5d in males (Figure S2B). Xist was increased in female *Tia1*^+/−^ mice by 83% (p = 0.019). Levels of Ddx3y, Uty, Eif2s3y, and Kdm5d were undetectable in females, as expected. Xist does not normally regulate genes on the Y chromosome. The responsiveness of these genes might reflect the higher levels of Xist expression in the *Tia1*^+/−^ mouse because previous studies have shown that elevated levels of Xist expression can lead to mislocalization of Xist binding (Pageau et al., 2007). In the case of our studies, we hypothesize that the reduced expression of Ddx3y, Uty, Eif2s3y, and Kdm5d results either from reduced transcriptional stimulation by TIA1, or from increased silencing due to increased Xist expression and concomitant ‘‘off target’’ repression of genes that share strong homology with the normal targets of Xist expression.

### An Unbiased Systems-Level Analysis Identifies Targets Driving Neurodegeneration in Tauopathy

Our transcriptomic analyses indicate that the behavioral protection from tauopathy in P301S *Tia1*^+/−^ mice is associated with a reversion to normal splicing of synaptic mRNAs (Figure 3). We next focused on the global, network-level changes underlying the aberrant transcriptional and splicing events detected in the RNA-seq, with the intention to discover biological targets capable of regulating the protective effects of TIA1 reduction. We used the NetDecoder algorithm previously developed by da Rocha et al. (2016) to systematically query the brain mRNA transcriptome of WT, P301S *Tia1*^+/−^, and P301S *Tia1*^+/+^ mice. NetDecoder is a network biology platform capable of reconstructing context-specific network profiles in terms of context-dependent information flow profiles using pairwise phenotypic comparative analyses (da Rocha et al., 2016). Here, information means the preference of flow mediated by protein-protein interactions due to shared biological functions, where the preference of information flow is determined by gene expression levels as well as coexpression coefficients of interacting proteins in a network. NetDecoder can therefore identify molecular interaction pathways to produce an unbiased and predictive ‘‘prioritized network’’ that mediates the differential information flows between WT, P301S *Tia1*^+/−^, and P301S *Tia1*^+/+^ mice, which thereby suggest biological targets for therapeutic intervention. A more detailed description of the NetDecoder algorithm is provided in the STAR Methods section.

NetDecoder analysis of the mRNA transcriptome of the WT, P301S *Tia1*^+/−^, and P301S *Tia1*^+/+^ cortex identified a prioritized network with various upstream network routers, downstream targets, and key genes (Figures 5A and S3). These key genes are not necessarily differentially expressed or alternatively spliced in the PS19 mice, but their functional importance is predicted to either regulate or be affected by the aberrant mRNA expression or splicing events. Many of the genes identified were already identified by NetDecoder as key genes in human AD versus age-matched control brain tissues, including APP, UBC, MYC, PARK2, MDM2, HDACs, TARDBP, RELA, HSP90, FYN, UBE2, and SUMO subunits, among others (Figures 5 and S3) (da Rocha et al., 2016). Our prioritized network from the transcriptome of the P301S *Tia1*^+/−^ versus P301S *Tia1*^+/+^ cortex successfully identified network routers and targets already known to regulate tau pathophysiology, such as the tau kinase FYN, the chaperone HSP90, and the E3 ubiquitin ligase STUB1/CHIP (Figure 5A), confirming the validity of this approach. More importantly, we also identified various routers, high-impact genes, and key target genes not previously associated with tau toxicity, including the proto-oncogene/transcription factor MYC and EGFR (Figures 5A–5C). Both of these proteins (MYC and EGFR) exhibited increased information flow in PS19 versus WT mice (Figure S3) and decreased information flow in the P301S *Tia1*^+/−^ compared to the P301S *Tia1*^+/+^ cortex (Figure 5B, blue). If these targets play a biologically important role, we hypothesized that inhibition of these proteins (which should mimic the decreased information flow exhibited in the P301S *Tia1*^+/−^ cortex) would be protective against tau-mediated neurodegeneration.

To investigate whether the genes highlighted by our prioritized network biology approach might mediate the neuroprotective effects in the P301S *Tia1*^+/−^ cortex, we proceeded to test whether functional reduction of these genes independent of TIA1 knockdown might also provide neuroprotection from SG-mediated toxicity. To do so, we assessed the effects of pharmacological inhibition of MYC, UBE2I, and EGFR on cell viability in primary mouse hippocampal neurons transduced with WT tau or vector control adeno-associated viruses (AAVs). Pharmacological inhibition of the SUMO-conjugating enzyme UBE2I was included because it demonstrated decreased information flow in P301S *Tia1*^+/−^ versus P301S *Tia1*^+/+^ mice, but not PS19 versus WT mice, and thereby served as a useful comparison for distinguishing tauopathy versus TIA1-specific effects. After 19 days in culture, neurons were treated with either vehicle control (DMSO, Veh) or chemical inhibitors to MYC (10058-F4, 5 µM), UBE2I (2-D08, 5 µM), or EGFR (TAG1478, 5 µM). After 30 min, the neurons were then treated for 1 h with sodium arsenite (125 µM) to induce SGs via the translational stress response, and toxicity was examined using both XTT and caspase 3 cleavage assays (Figures 6A–6C). Pharmacological inhibition of either MYC or EGFR indeed protected against toxicity in tau-overexpressing cultures, while inhibition of UB2EI provided no protection (Figures 6A and S3; similar results observed with both toxicity assays). MYC, EGFR, and UBE2I inhibition did not induce cell death in the absence of tau overexpression, as determined by the XTT and caspase cleavage assays (Figures 6 and S3). Although the predominant cell type in our primary hippocampal cultures are excitatory glutamatergic neurons, these cultures also contain glia, including astrocytes; thus, the neuroprotective mechanism underlying MYC and EGFR inhibition may occur in either the neurons or glia, or modulate the paracrine or physical interactions between both cell types. The effects of MYC (10058-F4, 1 or 10 µM), UBE2I (2-D08, 1 or 10 µM), or EGFR (TAG1478, 1 or 10 µM) inhibition on levels of TIA1 in SH-SY5Y cells were also examined by immunocytochemistry (Figures 6D and 6E). Pharmacological inhibition of MYC (1 µM only), EGFR, or UB2EI prevented increases in TIA1 associated with arsenite treatment (Figures 6D and 6E); the differential response of the MYC inhibitor 10058-F4 (active at 1 µM, inactive at 10 µM) might reflect a U-shaped response curve or off-target effects of the inhibitor at higher concentrations. Taken together, these results validate that many of the key genes detected in our unbiased systems biology NetDecoder analysis also regulate SG-mediated toxicity and RBP biology in primary cultured neurons, highlighting potential targets for therapeutic intervention.

### Comparison to Human AD Cases Suggests Overlapping Dysfunction in RNA Splicing

**Figure 4.**
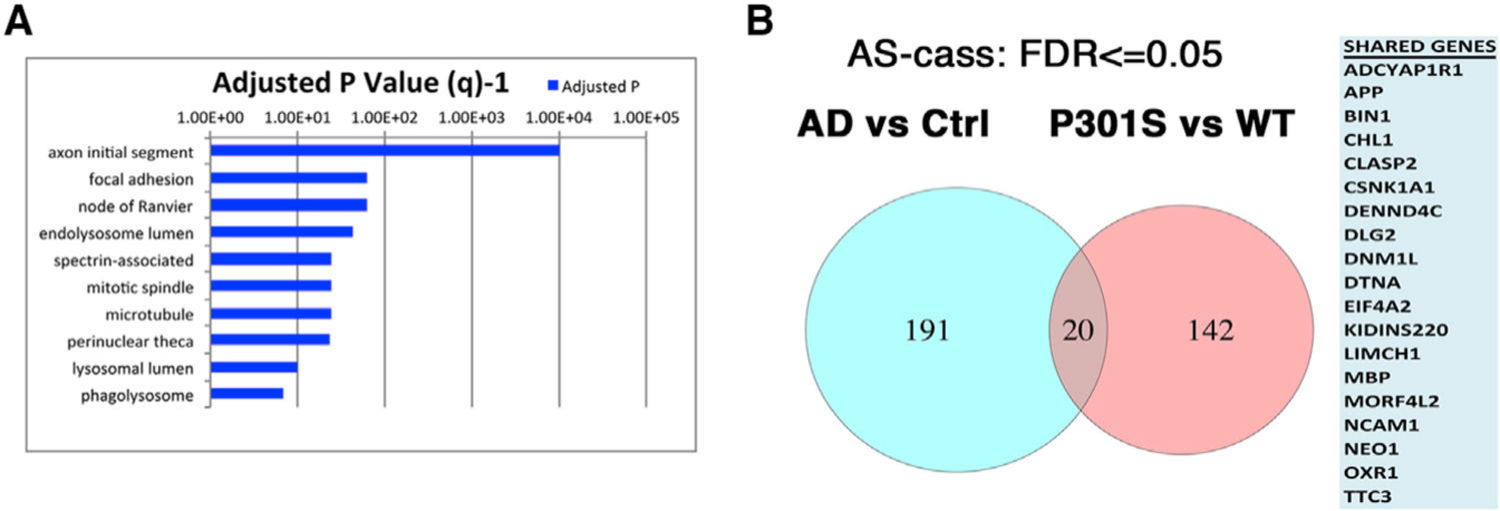
TIA1 Regulates the Expression of Sexually Dimorphic Genes (A) For human frontal cortex, top 10 most statistically significant Gene Ontology (GO) biological process annotation terms enriched (FDR < 0.05) in the gene list of mRNA transcripts exhibiting significant changes in splicing, as determined by OLego and Quantas splice variant analysis. (B) Venn diagrams highlighting the 20 genes are shared in common (at FDR < 0.05) between transcripts differentially spliced in the Alzheimer disease (AD) and PS19 mice. The right panel lists the 20 genes exhibiting overlap.

Many of the genes identified in our NetDecoder analysis were also previously identified as being key genes in human neurodegenerative diseases, including APP, PARK2, HTT, TARDBP, G3BP1, UBC, SETX, FYN, RELA, TP53, HDACs, UBE2I, and SUMO, among others (Ahn et al., 2009; Chen et al., 2004; Fernández-Nogales et al., 2014; Klein et al., 2000; Selkoe and Schenk, 2003; Um et al., 2012). These NetDecoder-identified key genes are those that exhibit significant changes in information flow in the whole biological network in the context of PS19 tauopathy, presumably due to aberrant RBP-mediated transcriptional or splicing events. Our NetDecoder analysis therefore suggested that there might be a common mechanistic link between the splicing defects observed in PS19 tauopathy and AD. We therefore investigated whether the defects observed in our PS19 tauopathy model might exhibit key overlap with the splicing defects observed in human AD. To probe whether the RNA-splicing changes we observed in the PS19 tau mouse brain showed a similar enrichment of neuronal genes in the AD brain, we downloaded data from the short read archive project SRP034831 of eight control and nine AD brain samples (Scheckel et al., 2016). Analysis of global splicing patterns in AD versus control brains showed large differences in splicing (Table S9; Figures 4A and 4B), confirming and extending suggestions from past work. Interestingly, analysis of the top 10 GO categories in AD samples showed some key points of overlap to those observed with the PS19 versus WT mice, with a prominence of neuronal (e.g., axon initial segment and nodes of ranvier) and vesicular categories (e.g., endolysosomal lumen, lysosomal lumen, and phagolysosome) (Figure 2A versus Figure 4A).

Analysis of the 211 genes showing significant cassette splice variants in the AD versus healthy control brain samples indicated that 72 out of the 211 genes (34.1%, p = 7.5E–06) exhibited TIA1 binding peaks in the iCLIP results (using comparisons to existing TIA1 iCLIP data) (Table S9, last tab) (Wang et al., 2010). We also specifically examined the presence of TIA1 binding sites for the 20 genes shared in common between the mouse (PS19 versus WT) and human (AD versus Control) datasets (Wang et al., 2010). We found that 11 out of the 20 genes exhibited TIA1 binding sites (FDR 8.2E–4, APP, CLASP2, CSNK1A1, DLG2, DNM1L, DTNA, LIMCH1, MORF4L2, OXR1, RPS24, and TTC3).

There were, however, clear distinctions between the datasets, potentially reflecting the different disease stages of the human versus mouse data (end stage post-mortem samples for human versus moderate disease for the mouse samples) and the differences between AD, a secondary tauopathy, versus our mouse model of primary tauopathy. The shared components are cogent, however, suggesting that the pathophysiology of AD and the PS19 tau mouse share an overall pattern of dysfunctional RNA splicing (Figure 4B), at least partly mediated by TIA1. This result also highlights the potential for therapeutic approaches targeting RBP aggregation and RNA metabolism in both primary tauopathies and secondary tauopathies such as AD.

## DISCUSSION

Our understanding of the pathophysiology of motor neuron disorders, including amyotrophic lateral sclerosis (ALS), has been revolutionized by major discoveries linking dysfunction of RBPs and RNA metabolism to the disease process. Mutations in RBPs and pathological accumulation of RBP aggregates are associated with ALS, frontotemporal dementia (FTD), and myopathies (Couthouis et al., 2011, 2014; Hackman et al., 2013; Kwiatkowski et al., 2009; Neumann et al., 2006, 2009; Taylor et al., 2016; Vance et al., 2009; Mackenzie et al., 2017; Yuan et al., 2018). Although most tau is associated with axonal tubulin, in diseases such as AD, tau accumulates in the somatodendritic arbor. Recent evidence indicates that somatodendritic tau functions in part to regulate SG accumulation and the translational stress response (Brunello et al., 2016; Meier et al., 2016; Vanderweyde et al., 2016). The discovery that the association of tau with RNA promotes liquid-liquid phase separation (LLPS) provides a biophysical mechanism that links the physiological process governing formation of SGs and other membrane-less organelles with the pathophysiological process producing tau aggregation and neurodegeneration (Feric et al., 2016; Li et al., 2012). The work presented above demonstrates that modulation of RBP aggregation by TIA1 reduction partially rescues the dysregulation of RNA splicing that is associated with tauopathy in PS19 mice.

Transposon expression showed no significant differences in our sample sets, which highlights the selectivity of the dysfunctional RNA metabolic processes in specific neurodegenerative diseases (for example, transposon expression in ALS and mRNA splicing in tauopathies). Our results differ from a recent report by Guo et al. (2018), which concluded that transposon activation occurs in AD. The difference in our conclusions lies in the statistical analysis. The Salmon tool used by Guo et al. assumes that the expression of total transposable elements is proportional to the total transcriptome size. However, in situations where transcriptional expression in one sample differs greatly from another sample (such as being lower), the algorithm artificially ‘‘stretches’’ the transposal element expression for the smaller sample up to the scale of the other sample, which makes some transposable elements appear to be artificially elevated. Therefore, we applied the Salmon algorithm after correcting for differences in the number of transposable element reads and confirmed that our data did not identify any transposons that exhibit statistically different levels of expression in our samples. These results demonstrate that the dysregulation of RNA metabolism in PS19 mice is specific to changes in mRNA transcription and alternative splicing, and suggests that aberrant mRNA splicing occurs in tauopathies much like it does in other diseases associated with RBP mutations or aggregation (Prudencio et al., 2015). More importantly, the results from this study and others place tauopathy in the continuum of diseases associated with dysfunction of RBPs.

Our analyses also showed similar trends in splicing dysfunction in the PS19 mice and human AD brain. 70% of the genes that show differential splicing in both PS19 mice and human AD brain also exhibit either genetic linkage to AD (15%) (Kohli et al., 2016; Rosenberg et al., 2016) or demonstrable involvement in disease processes (55%) (Finelli et al., 2015; Fujioka et al., 2013; Gamir-Morralla et al., 2017; Hanger et al., 2007; López-Menéndez et al., 2013). Prior studies have demonstrated dysfunction of RNA splicing in ALS, a neurodegenerative movement disorder that shows strong genetic associations with mutations in RBPs (Prudencio et al., 2015). The importance of our results is further supported by a prior study documenting splicing dysfunction in AD brains (Bai et al., 2013; Barbash et al., 2017), as well as work that specifically examined the function of ELAV proteins (such as HuR) and observed a similarly strong dysfunction of RNA splicing (Scheckel et al., 2016). Our study extends beyond those past findings by also examining GSEA, highlighting the prominence of spliced transcripts important for neuronal/synaptic activity in the AD brain.

The striking role of RNA-splicing dysfunction in the pathophysiology of tauopathies opens avenues for potential therapeutic intervention. The spliceosome complex utilizes RBPs and is regulated at the level of post-translational modifications of RBPs, as well as direct modifications to RNA and DNA. Many of the RBPs associated with the spliceosome aggregate in AD and mouse models of tauopathy, some co-localizing with tau inclusions, such as TIA1, and others forming independent inclusions, such as TDP-43, U1–70K, and G3BP1 (Amador-Ortiz et al., 2007; Arai et al., 2009; Bai et al., 2013; Bigio et al., 2010; Vanderweyde et al., 2012, 2016). We recently demonstrated that TIA1 reduction protects against the neurodegeneration associated with tauopathy (Apicco et al., 2018; Vanderweyde et al., 2016). We now demonstrate that this protection is associated with a corresponding rescue of aberrant RNA splicing. While the relative contributions of direct TIA1 function, differential RBP expression, and altered proteostasis or SG dynamics remain to be determined, the ability of TIA1 reduction to rescue the splicing dysfunction demonstrates the importance of RBP biology in the pathophysiology of tauopathies.

**Figure 5.**
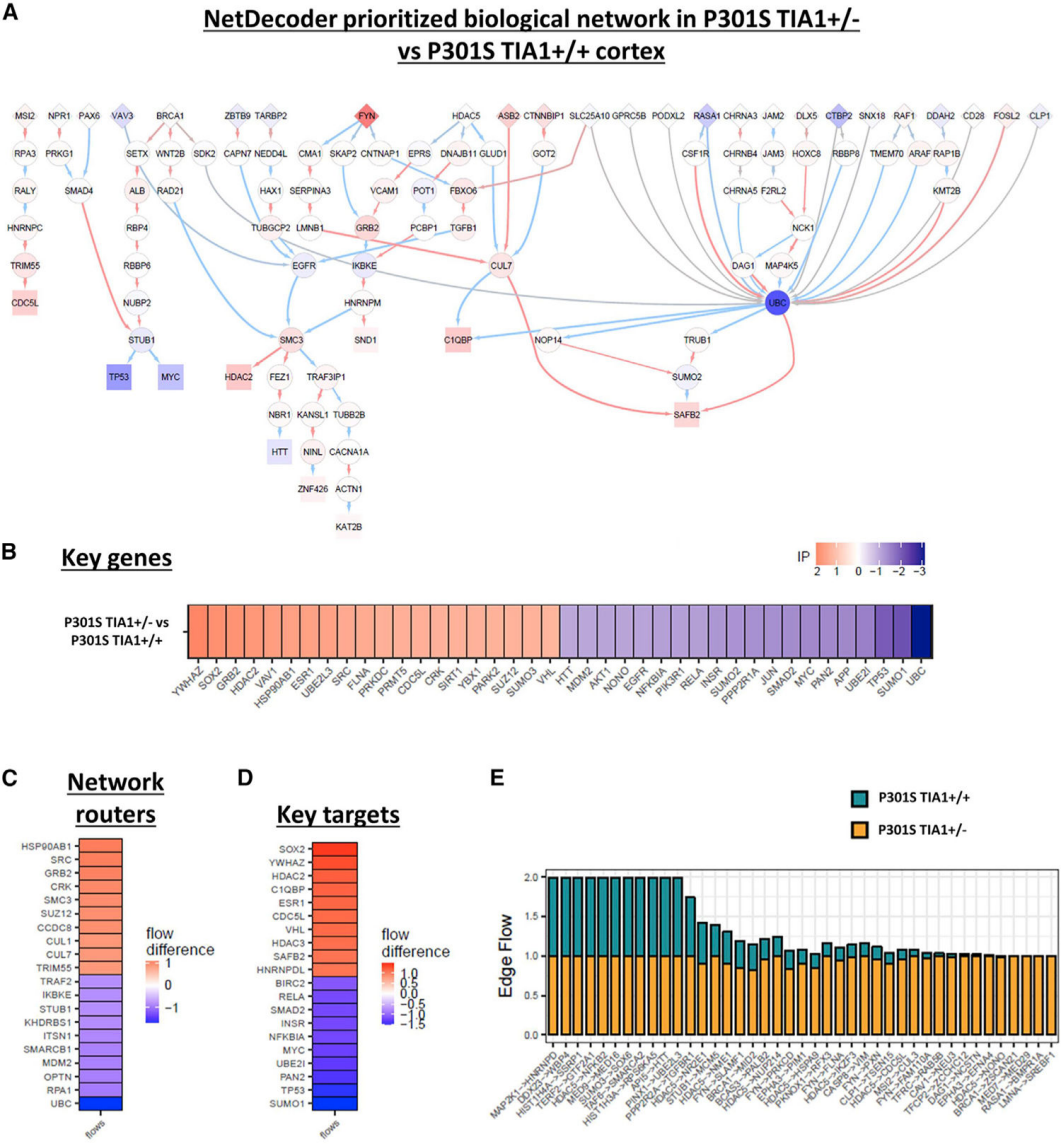
Disease Phenotype in Neuroprotected PS19 Mice Is Regulated by a Prioritized Biological Network NetDecoder analysis was performed on the RNA-seq data from 9-month PS19 *Tia1*^+/+^ and P301S *Tia1*^+/−^ cortex in order to identify context-dependent changes inherent to the disease phenotype, as described previously (da Rocha et al., 2016). (A) Prioritized network consisting of biological pathways predicted to regulate the differences in phenotype between P301S *Tia1*^+/−^ (protected) and PS19 *Tia1*^+/+^ (tauopathy) mice. Red and blue nodes denote proteins exhibiting either increased (red) or decreased (blue) information flow in P301S *Tia1*^+/−^ compared to PS19 *Tia1*^+/+^ cortex. Arrows denote direction of information flow from source genes to target genes based on known protein-protein interaction (PPI) data. Network routers (diamonds) are upstream of intermediary (circles) and target (square) protein nodes. (B–D) Key genes (B), network routers (C), and key targets (D) identified by NetDecoder analysis to mediate context-dependent disease phenotype. (E) Selected protein-protein interactions (edges) showing differences in information flows between *Tia1*^+/−^ and *Tia1*^+/+^ contexts.

**Figure 6.**
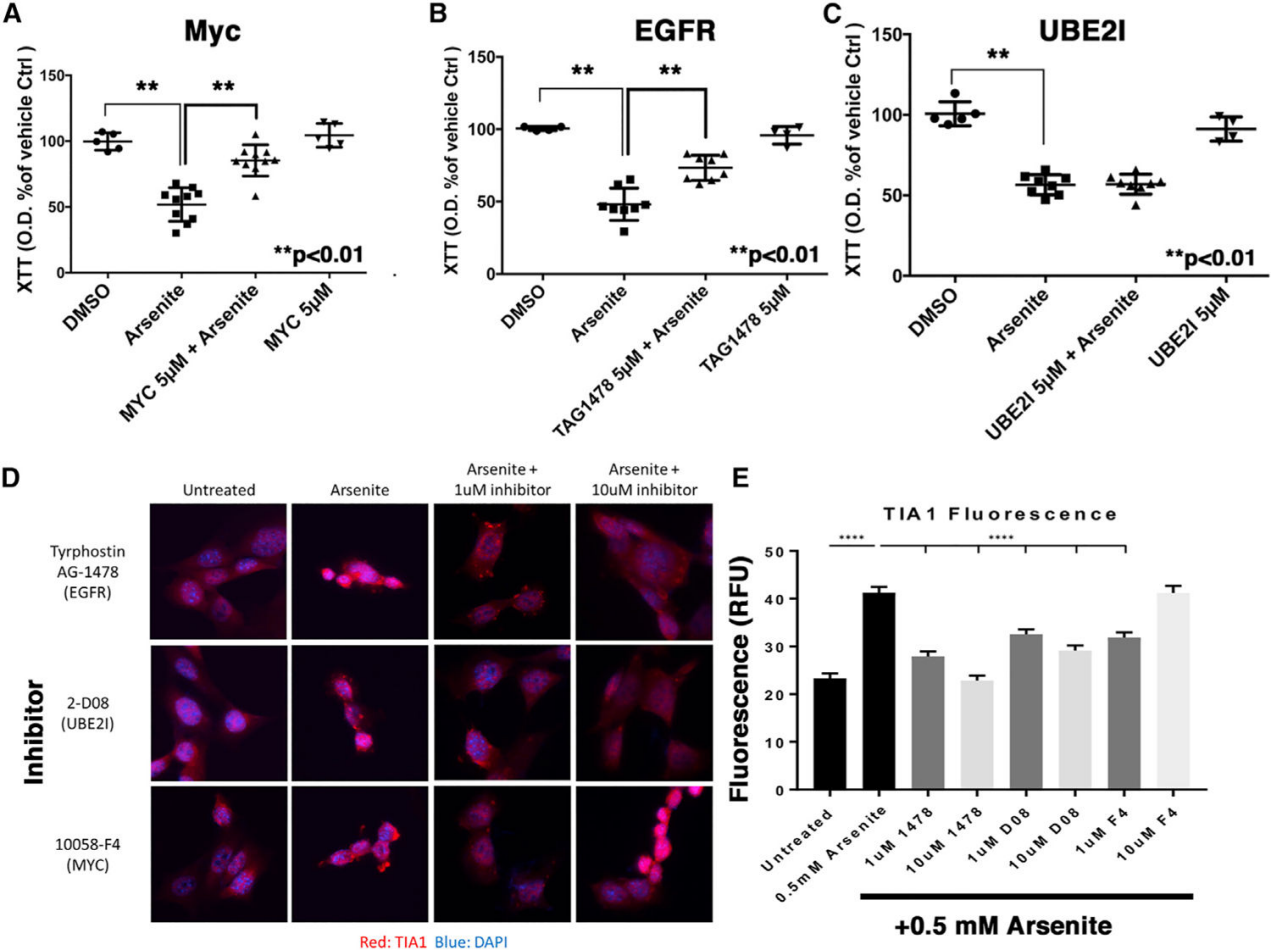
Inhibition of MYC and EGFR Protects against Tau-Mediated Toxicity DIV14 cultures of hippocampal mouse neurons (C57BL/6J) transduced with AAV2 WT human 1N4R tau were treated with inhibitor for 1 h, then arsenite (125 µM) was added, and assayed for viability after 12 h. (A–C) (A) MYC inhibitor (10058-F4, 5 µM), (B) EGFR (TAG1478, 5 µM), and (C) UBE2I inhibitor (2-D08, 5 µM). **p < 0.0001, n = 4–8. (D and E) SH-SY5Y cells were pre-treated with MYC inhibitor (10058-F4, 1, 10 µM), EGFR (TAG1478, 1, 10 µM), and UBE2I inhibitor (2-D08, 1, 10 µM), then exposed to arsenite, fixed, and labeled with anti-TIA1 antibody. Representative images (D) and quantification of TIA1 immunofluorescence levels (E) are shown. Each of the inhibitors prevented arsenite-induced increases in TIA1 levels (E).

To further elucidate the global pathological impact of dysregulated RNA splicing in the context of PS19 taoupathy, we used an unbiased systems biology approach to gain broad insight into the mechanisms of neuroprotection afforded by TIA1 reduction. The NetDecoder analysis of the brain mRNA transcriptome in P301S *Tia1*^+/−^ versus P301S *Tia1*^+/+^ mice generated a list of key genes including network routers and key targets predicted to drive the neuroprotection associated with TIA1 reduction (Figure 5) (da Rocha et al., 2016). The NetDecoder-prioritized network indicates that P53 and MYC operate in parallel pathways that act downstream of BRCA1 (Figure 5A). These genes are most classically associated with DNA damage (and caspase activity for P53), but the pathway is notable for including SETX, which has mutations that are associated with motor neuron diseases (Chen et al., 2006; Couthouis et al., 2011, 2014). We also note that the expression of BRCA1 and Xist are inversely correlated in breast cancer cell lines, raising the possibility that changes in BRCA1 activity might underlie the increased Xist expression in the TIA1^+/−^ cells (Sirchia et al., 2009). A recent study of immune cells also supports the role of TIA1 in regulating p53 and demonstrates that the regulation occurs post-transcriptionally (Díaz-Muñoz et al., 2017). Targeting the EGFR is also of interest because the levels of EGFR transcript are altered in AD, suggesting involvement in the disease process (Conejero-Goldberg et al., 2011). Our experimental studies confirmed that inhibiting MYC or EGFR indeed resulted in protection against SG-mediated degeneration in tau-overexpressing primary neurons (Figure 6), suggesting a role for these pathways in the neurodegenerative process. The ability of these pathways to prevent stress-induced increases in TIA1 implicates regulation of TIA1 in the mechanisms of action (Figure 6). The lack of neuroprotective benefit observed with the UBE2I inhibitor might indicate that UBE2I is protective. Polymorphisms in the SUMO ligase UBE2I are genetically associated with AD in a Korean cohort, but studies suggest that sumoylation is protective in brain injury (Ahn et al., 2009; Yang et al., 2016). Thus, the upregulation of UBE2I in the PS19 mouse might be a compensatory response to the neurodegenerative changes or a tauopathy-independent effect of *Tia1* heterozygosity.

This report demonstrates that tauopathy is associated with a profound dysregulation of RNA splicing. We also identify a number of candidate therapeutic targets that mediate the neuroprotective benefits associated with TIA1 reduction. The potential value of targeting RNA metabolism is highlighted by a recent study using a TDP-43 model of ALS, which demonstrated that reducing the RBP ataxin-2 also produced strong rescue of the disease phenotype (Becker et al., 2017). These studies present RBPs and RNA metabolism as powerful modulators of disease progression in both tauopathies and motor neuron disorders. This study also demonstrates that the effects of dysfunctional RNA splicing reach far from the soma, contributing to the dysfunctional neurotransmission that is associated with neural network dysfunction in tauopathies. Finally, deciphering context-specific information flows in a biological network using our unbiased systems biology approach reveals a wide range of therapeutic candidates for the treatment of tauopathies.

## STAR★METHODS

### LEAD CONTACT AND MATERIALS AVAILABILITY

Further information and requests for resources and reagents should be directed to and will be fulfilled by the Lead Contact, Benjamin Wolozin, M.D., Ph.D. (bwolozin@bu.edu). This study did not generate unique reagents.

### EXPERIMENTAL MODEL AND SUBJECT DETAILS

#### In Vivo Animal Subjects

Tia1−/− mice (B6.129S2(C)-Tia1tm1Andp/J) were generated by Anderson and colleagues and obtained from Harvard University, Dana Farber Cancer Institute (Phillips et al., 2004); these mice have been backcrossed for 10+ generations to a 100% C57BL/6J genetic background. PS19 (B6;C3-Tg(Prnp-MAPT*P301S)PS19Vle/J, stock #008169) and C57BL/6J (stock #000664) mice were purchased from The Jackson Laboratory (Yoshiyama et al., 2007). All animals were housed in IACUC-approved vivariums at Boston University School of Medicine. To generate colonies of P301S Tia1+/+ and P301S Tia1+/− mice, PS19 mice were bred with Tia1−/− mice to produce pups that were heterozygous for the endogenous mouse Tia1 allele (Tia1+/−) and either transgenic (P301S+/−, or ‘P301S Tau’) or non-transgenic (P301S−/−, or ‘WT Tau’) for human P301S Mapt. Transgenic (P301S+/− Tia1+/−) and non-transgenic (P301S−/− Tia1+/−) pups from the F1 generation were then bred to produce littermate control P301S Tia1+/+ and P301S Tia1+/− mice on identical genetic backgrounds, which were then used in the experiments described in this manuscript. Mice were aged to 9 months of age and euthanized for RNA extraction and cDNA library preparation, as described below. Only male mice were used in the analysis, except where noted otherwise. Mice that were displaying end-stage and/or neuromuscular symptoms of disease (postural instability, dragging of one or more hind limbs, or greater than 10 percent loss in body weight) were excluded from the RNA-seq analysis. Animals were assigned to experimental groups by genotyping, as described below. The animal care and use committee for Boston University Medical Center approved the animal protocols used in this manuscript.

#### Human Subjects

Data downloaded from the short read archive project SRP034831

#### Primary neuron cultures

Primary neurons used in these studies were generated from mixed sex neonatal C57BL/6J mouse pups. Pups were anesthetized, euthanized, and their hippocampi dissected in Hank’s Buffered Saline Solution (HBSS). Cells were plated in Poly-D-Lysine coated cell culture plates and maintained in Neuron Feeding Media (Neurobasal media supplemented with 1% penicillin- streptomycin, B-27, and L-Glutamine) for 14 to 21 days *in vitro* (DIV). Neuron viability was then analyzed by the methods described below.

#### SH-SY5Y Cell Culture

SH-SY5Y cells were obtained from ATCC (CRL-2266) in DMEM/F12 medium with 10%FBS. SH-SY5Y cells were originally derived from a human female.

#### Sex of Model Systems

P301S Mapt mice were male, unless otherwise noted

Primary neuronal cultures were mixed sex

SH-SY5Y cells are female

### METHOD DETAILS

#### Genotyping

Tail snips from ear tagged mice were digested using Proteinase K, and the DNA was purified using the QIAGEN DNeasy kit according to manufacturer’s instructions (cat# 69504). The human 1N4R P301S transgene was amplified using the following primers (hTau Forward = GGG GAC ACG TCT CCA CGG CAT CTC AGC AAT GTC TCC; hTau Reverse = TCC CCC AGC CTA GAC CAC GAG AAT), using the cycling parameters specified by The Jackson Laboratory (https://www.jax.org/strain/008169CODE:25393,008169). Mice were also genotyped for both the wild-type Tia1 allele (WT mTia1 Forward = CTC CTT TAC CAG GAC CAC CA; WT mTia1 Reverse = ACC ATG GGG AAA AGG AGG TA) and frame shifted mutant allele that does not encode TIA1 protein (Mutant mTia1 Forward = CTC CTT TAC CAG GAC CAC CA; Mutant mTia1 Reverse = GCC AGA GGC CAC TTG TGT AG), also using the cycling parameters specified by The Jackson Laboratory (https://www.jax.org/strain/009248CODE:23469,009248). Amplified DNA was separated by electrophoresis in a 2% agarose gel, and visualized with 1% ethidium bromide using a BioRad imager.

#### RNA-Sequencing and Analysis

Total brain (cortex) RNA extracts were purified according to manufacturer’s instruction using the QIAGEN Qiazol Lysis Reagent (Cat# 79306) and RNeasy Mini kit (Cat# 73404). The quality of the RNA preparations was confirmed using an Agilent bioanalyzer instrument (RIN scores > 9.0). A total of 100 ng of RNA in a 5 ml volume was used for library preparation and RNA-Seq (n = 3/genotype). Paired-end sequencing libraries were prepared by the Mayo Clinic sequencing core facilities (Rochester, MN) with the TruSeq Stranded Total Sample Preparation kit (Illumina), after which samples were subjected to quality control, cluster generation, and sequencing at a read depth of 60 million reads per sample on the Illumina HiSeq 2000 platform. The reads were de-multiplexed and converted to FASTQ format using CASAVA software from Illumina (by the Mayo Clinic core). All RNA-Seq data for each individual RNA sample are available at the NCBI Gene Expression Omnibus under a unique accession number (to be provided upon acceptance of the manuscript).

#### Sequence Alignment

Sequence alignment was performed using Bowtie and TopHat v2.0.12 programs against the UCSC mm10 Assembly (Kim et al., 2013). Expression values were calculated with featureCounts v1.4.6-p2, and differential expression analysis was done using DESeq2 (Love et al., 2014), and the volcano plots were made in R (v3.1.1; http://www.r-project.org/) (Langmead et al., 2009; Liao et al., 2014; Trapnell et al., 2012). Gene Set Enrichment Analysis (GSEA) was performed as described previously (Subramanian et al., 2005). Splicing analysis: Quantification of alternative splicing (AS) events were done separately using OLego, a seed-and-extend aligner that has high sensitivity for splice-junction mapping of very small seeds (14-nt seed size) (Wu et al., 2013). After alignment using OLego, we determined and quantified differential cassette exon events using the Quantas module, as described in OLego, and used Fisher’s exact test to evaluate the statistical significance of splicing changes. For analysis of iCLIP data for human TIA1 (Wang et al., 2010), reads were mapped to reference genome UCSC hg19 using Bowtie v1.1.0 after removing barcodes and then peaks were called using MACS v1.4.2 (Langmead et al., 2009; Zhang et al., 2008).

#### Quantitative RT-PCR

Total brain (cortex) RNA extracts purified as described above were reverse-transcribed using the Applied Biosystems High-Capacity cDNA Reverse Transcription Kit according to manufacturer’s instruction (Cat# 4368814). RT-PCR reactions were performed in quadruplicate in a 384-well plate using 1 ng cDNA template, 200 nM forward and reverse primers, and 2x iQ SYBR Green Supermix (BioRad, Cat# #1708880) per well. Primer pairs for splice isoforms were designed to span exon-junctions of alternative splicing events. RT-PCR reactions were performed on a 7900HT Fast Real-Time PCR System (Applied Biosystems). The primers used were the following: Gria2_total_forward, GTATCCTTCATCACACCAAG; Gria2_total_reverse, CTCAATCAAGCTAAGGAGTG; Gria2_Ex14_Flip_forward, TAAGAAATGCGG TTAACCTC; Gria2_Ex14_Flip_reverse, GTCGTACCACCATTTGTTT; Gria2_Ex15_Flop_forward, GTCCACAATGAATGAGTACA; Gria2_Ex15_Flop_reverse, GCACTGGTCTTTTCCTTAAT; Snap25_total_forward, TTGCATTGAAGAAGAAACCT; Snap25_total_reverse, AGAACCTTGTCTTCTCCG; Snap25_Ex5_Incl_forward, AAGAAGGCATGAACCATATC; Snap25_Ex5_Incl_reverse, ACAAGGACATA TGAAAAGGC, Snap25_Ex6_Incl_forward, TTTGGTTATGTTGGATGAGC; Snap25_Ex6_Incl_reverse, ATCCTGATTATTGCCCCAG; Camk2b_total_forward, GTATGCAGCCAAGATCATTA; Camk2b_total_reverse, AGCTTCTCTCTCCAGTTTC; Camk2b_Ex13_Skip_forward, TGGAATGTCTGAAGAAGTTC; Camk2b_Ex13_Skip_reverse, TCTGGGGCTGAGAAATTC; Camk2b_Ex13_Incl_forward, GAAA GCAGATGGAGTCAAG; Camk2b_Ex13_Incl_reverse, CTCTATGGTTGTGTTGGTG; Gapdh_total_forward, TGGTCTACATGTTCCA GTAT; and Gapdh_total_reverse, GATGACAAGCTTCCCATTC. The relative amounts of each transcript, including splice isoforms, were calculated by normalizing the Ct scores to a reference transcript (∆DCtGapdh). Alternative splice isoforms were then further normalized to the total transcript level detected in the sample. The ratio of exon inclusion was then determined to be [(Isoform 1 ∆DCtGapdh) / (Total ∆DCtGapdh)] / [(Isoform 2 ∆DCtGapdh) / (Total ∆DCtGapdh)]. Since the raw Ct counts better reflect the amplification efficiency of each primer pair than the true expression levels of each splice isoform, we normalized the relative exon inclusion ratios in each genotype to WT level = 1. Thus, the values reported in the alternative splicing figures accurately reflect the relative changes in exon inclusion between groups, but not necessarily their true physiological levels in each genotype. RT-PCR reactions were performed using 3 male and 3 female tissues per genotype (n = 6/group) to control for sex differences in alternative splicing events and transcript expression.

#### Context-Dependent Network Analysis Using NetDecoder

NetDecoder is a network biology platform which uses process-guided flow algorithm to model context-specific information flows based on interactions between genes at protein level for a given biological state and perform differential network analysis between a pair of biological phenotypes (da Rocha et al., 2016). In current study, pair of interested phenotypes to compare is PS19 P301S Tia1 ± versus PS19 P301S Tia1 +/+, where context-dependent weighted interactions for each phenotype, source genes and targets genes were generated as below.

Context-dependent weighted interactome: Raw gene counts were normalized using DESeq2 (Love et al., 2014) and then mapped to their human homolog using the Mouse Genome Informatics database (Blake et al., 2017). Pearson’s coefficients of gene expression values were calculated across samples within each of the PS19 P301S Tia1 ± and PS19 P301S Tia1 +/+ groups for pairwise gene interactions available in a comprehensive pairwise protein-protein interaction (PPI) network, as described in da Rocha et al. (2016). The absolute values of the Pearson’s coefficients were used as weights of the PPI interaction network, forming context-specific weighted interactomes for each group. The Pearson’s coefficients were then used to define edge capacity and cost for flow calculation.

Source genes: These are genes where flows begin. Because we are interested in gene candidates that might offer neuroprotection in P301S Tia1 ± mice, the source genes were identified using a template-matching approach (Pavlidis and Noble, 2001) to select genes which are highly expressed in PS19 P301S Tia1 ± group but low in PS19 P301S Tia1 +/+ group. Genes were ranked by template-matching score in descending order and top 0.5% of the genes were selected as source genes.

Target genes: These are genes where flows end. The target genes were defined as transcriptional regulators based on GO annotations, i.e., genes of GO:0003676 (nucleic acid binding), GO:0006355 (regulation of transcription, DNA-templated) and GO:0008134 (transcription factor binding).

With the above setup and data preparation, NetDecoder was then used to find subnetworks with maximum flow from source to target genes mediated via GO annotations in each gene in the PPI network through the context-specific weighted interactomes for P301S Tia1 ± and PS19 P301S Tia1 +/+ groups respectively. More detailed description of process-guided flow algorithm can be found in da Rocha et al. (2016). By comparing the resulting flow profiles in PS19 P301S Tia1 ± and PS19 P301S Tia1 +/+ subnetworks, genes with large flow difference in the 2 flow subnetworks were identified as network routers when they were intermediary nodes in the context-specific weighted interactome and as key targets when they were target nodes. High impact score, which was a scoring scheme taking into consideration flow differences, establishment of new flows and change of sign of gene expression correlations (i.e., from positive Pearson correlation coefficient to negative coefficient or vice versa), were used to rank the genes and identify high impact genes. Prioritized subnetwork (Figure 5A), which was a collection of flow paths with at least any 2 categories of genes out of network routers, key targets and high impact genes, was established by comparing the 2 flow subnetworks. Heatmaps of high impact genes, network routers and key targets (Figures 5B–5D) as well as bar charts for edges with large flow differences (Figure 5E) were plotted using the ggplot2 package (Wickham, 2009) in R. The prioritized subnetwork was visualized using Cytoscape (Cline et al., 2007)

#### Cell Culture

Hippocampi from neonatal C57BL/6J mice were dissected in HBSS, triturated, and cultured for 21 days *in vitro* (DIV), as described previously (Vanderweyde et al., 2016). Cells were plated in either 96-well plates (5,000 neurons/well; for caspase activation assay) or 12 mm glass coverslips previously coated with 100 ng/ml Poly-D-Lysine. Neurons were maintained in neuronal feeding media (Neurobasal media supplemented with 1% penicillin-streptomycin, B- 27, and L-Glutamine), which was replaced with 1/3 volume per well twice per week (every 3–4 days). On DIV 2, neurons were transduced with AAVs (serotype 1, generously provided by the laboratory of Dr. Leonard Pettrucelli) expressing WT 0N4R human Tau (WT Tau AAV1), or vector control (Ctrl AAV1) at a multiplicity of infection (MOI) of 200. On DIV 14, neurons were treated with chemical inhibitors to MYC (5 µM 10058-F4, cat# F3680, Sigma), UBE2I (5µM 2-D08, cat# SML1052, Sigma), or EGFR (5 µM, Tyrphostin AG-1478, cat#T4182, Signa) for 48 h. Then the cells were treated with inhibitor for 1hr, then sodium arsenite (125 µM, final concentration) was added. After 12 more hours, XTT reagent was added and viability measured after 4 hr. The experiments were done in a similar manner for the caspase activity assays, except that arsenite was not added; caspase activity was measured with the Casp 3/7 Glo caspase activation kit (Promega) on DIV 21. For immunocytochemistry experiments, SH-SY5Y cells were treated with each inhibitor (doses of 1 or 10 µM) for 30 min, then treated for 45 min with a combination of the inhibitor plus 0.5 mM arsenite. The cells were then fixed, and immunocytochemistry for TIA1 (anti-TIA1 antibody Abcam 40693, 1:200 dilution) was done.

### QUANTIFICATION AND STATISTICAL ANALYSIS

Sequence alignment was performed using Bowtie and TopHat v2.0.12 programs against the UCSC mm10 Assembly (Kim et al., 2013). Expression values were calculated with feature Counts v1.4.6-p2, and differential expression analysis was done using DESeq2 (Love et al., 2014)

Splicing analysis: Quantification of alternative splicing (AS) events were done separately using OLego, a seed-and-extend aligner that has high sensitivity for splice-junction mapping of very small seeds (14-nt seed size) (Wu et al., 2013). After alignment using OLego, we determined and quantified differential cassette exon events using the Quantas module, as described in OLego, and used Fisher’s exact test to evaluate the statistical significance of splicing changes.

For all neuron culture and RT-PCR validation experiments, statistical analysis was performed using GraphPad Prism software. All statistical tests used, exact p values, and sample sizes are described in the figure legends.

### DATA AND SOFTWARE AVAILABILITY

The RNaseq data have been deposited in the GEO under accession ID code GSE109226.

## Supplementary Material

1

8

9

10

11

2

3

4

5

6

7

## Figures and Tables

**Table:** KEY RESOURCES TABLE

REAGENT or RESOURCE	SOURCE	IDENTIFIER
Chemicals, Peptides, and Recombinant Proteins		
MYC inhibitor (10058-F4)	Sigma	Cat# F3680
UBE2I inhibitor (2-D08)	Sigma	Cat# SML1052
EGFR inhibitor (Tyrphostin AG-1478)	Sigma	Cat# T4182
Critical Commercial Assays		
Casp 3/7 Glo caspase activation kit	Promega	G8093
XTT assay	Sigma-Aldrich	11465015001ROCH E
iQ SYBR Green Supermix	BioRad	Cat# 1708880
High-Capacity cDNA Reverse Transcription Kit	Applied Biosystems	Cat# 4368814
TruSeq Stranded Total Sample Preparation kit	Illumina	Cat# 20020596
RNeasy Mini kit	QIAGEN	Cat# 73404
Deposited Data		
RNA-seq data	GEO	GSE109226
Experimental Models: Organisms/Strains		
PS19 mice	The Jackson Laboratory	Stock #008169 RRID: IMSR_JAX:008169
C57BL/6J mice	The Jackson Laboratory	Stock #000664 RRID: IMSR_JAX:000664
B6.129S2(C)-Tia1tm1Andp/J	Anderson Laboratory	RRID:MGI:3037319
Software and Algorithms		
Tophat 2.0.12	Kim et al., 2013	http://ccb.jhu.edu/software/tophat/
featureCounts v1.4.6-p2	Liao et al., 2014	http://subread.sourceforge.net/
DESeq2	Love et al., 2014	https://bioconductor.org/packages/release/bioc/html/DESeq2.html
Gene Set Enrichment Analysis	Subramanian et al., 2005	http://software.broadinstitute.org/gsea/index.jsp
OLego	Wu et al., 2013	https://zhanglab.c2b2.columbia.edu/index.php/OLego
Bowtie 1.1.0	Langmead et al., 2009	http://bowtie-bio.sourceforge.net/index.shtml
MACS 1.4.2	Zhang et al., 2008	http://liulab.dfci.harvard.edu/MACS/
NetDecoder	da Rocha et al., 2016	http://netdecoder.hms.harvard.edu/

## Highlights

- Transcripts encoding spliceosomal and mRNA binding proteins are reduced in PS19 brain
- Alternative splicing in PS19 brain favors transcripts encoding synaptic proteins
- TIA1 regulates the expression of Xist and sexually dimorphic genes
- Inhibition of MYC and EGFR protects against tau-mediated toxicity
