# Supplementary material for: Dysregulation of RNA Splicing in Tauopathies
Source: Cell Rep. Author manuscript; Available in PMC 2020 Jan 3. (PMC6941411; doi:10.1016/j.celrep.2019.11.093)
Supplement: 1 [file NIHMS1547525-supplement-1.pdf]

Cell Reports, Volume *29*

## Supplemental Information

# Dysregulation of RNA Splicing in Tauopathies

**Daniel J. Apicco, Cheng Zhang, Brandon Maziuk, Lulu Jiang, Heather I. Ballance, Samantha Boudeau, Choong Ung, Hu Li, and Benjamin Wolozin**

# Supp. Fig. 1

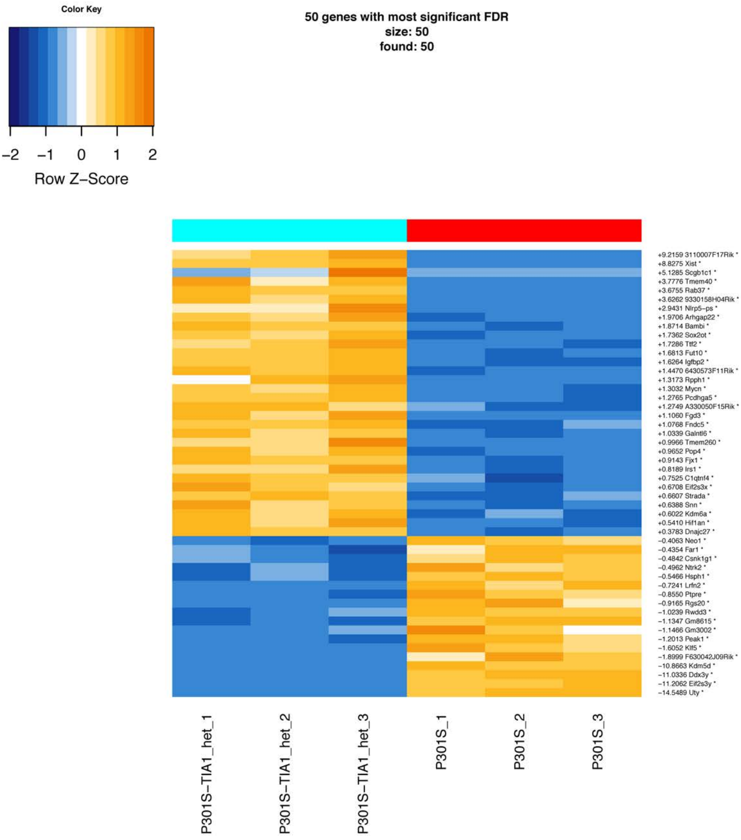
Color Key
−2
−1
0
1
2
Row Z−Score
50 genes with most significant FDR
size: 50
found: 50
+9.2159 3110007F17Rik *
+8.8275 Xist *
+5.1285 Scgb1c1 *
+3.7776 Tmem40 *
+3.6755 Rab37 *
+3.6262 9330158H04Rik *
+2.9431 Nlrp5−ps *
+1.9706 Arhgap22 *
+1.8714 Bambi *
+1.7362 Sox2ot *
+1.7286 Ttf2 *
+1.6813 Fut10 *
+1.6264 Igfbp2 *
+1.4470 6430573F11Rik *
+1.3173 Rpph1 *
+1.3032 Mycn *
+1.2765 Pcdhga5 *
+1.2749 A330050F15Rik *
+1.1060 Fgd3 *
+1.0768 Fndc5 *
+1.0339 Galntl6 *
+0.9966 Tmem260 *
+0.9652 Pop4 *
+0.9143 Fjx1 *
+0.8189 Irs1 *
+0.7525 C1qtnf4 *
+0.6708 Eif2s3x *
+0.6607 Strada *
+0.6388 Snn *
+0.6022 Kdm6a *
+0.5410 Hif1an *
+0.3783 Dnajc27 *
−0.4063 Neo1 *
−0.4354 Far1 *
−0.4842 Csnk1g1 *
−0.4962 Ntrk2 *
−0.5466 Hsph1 *
−0.7241 Lrfn2 *
−0.8550 Ptpre *
−0.9165 Rgs20 *
−1.0239 Rwdd3 *
−1.1347 Gm8615 *
−1.1466 Gm3002 *
−1.2013 Peak1 *
−1.6052 Klf5 *
−1.8999 F630042J09Rik *
−10.8663 Kdm5d *
−11.0336 Ddx3y *
−11.2062 Eif2s3y *
−14.5489 Uty *
P301S−TIA1_het_1
P301S−TIA1_het_2
P301S−TIA1_het_3
P301S_1
P301S_2
P301S_3

**Supplementary Figure 1 (related to Figures 2 and 3): HEAT map comparing gene expression of the PS19 P301S *Tia1+/+ vs* PS19 P301S *Tia1+/-* mice.** Comparison of transcriptional profiles of 9-month P301S *Tia1*+/+ and P301S *Tia1*+/- mice. The transcripts with the 50 top FDR scores were selected for presentation. The corresponding data are listed in Supplemental Table 7.

Supp. Fig. 2

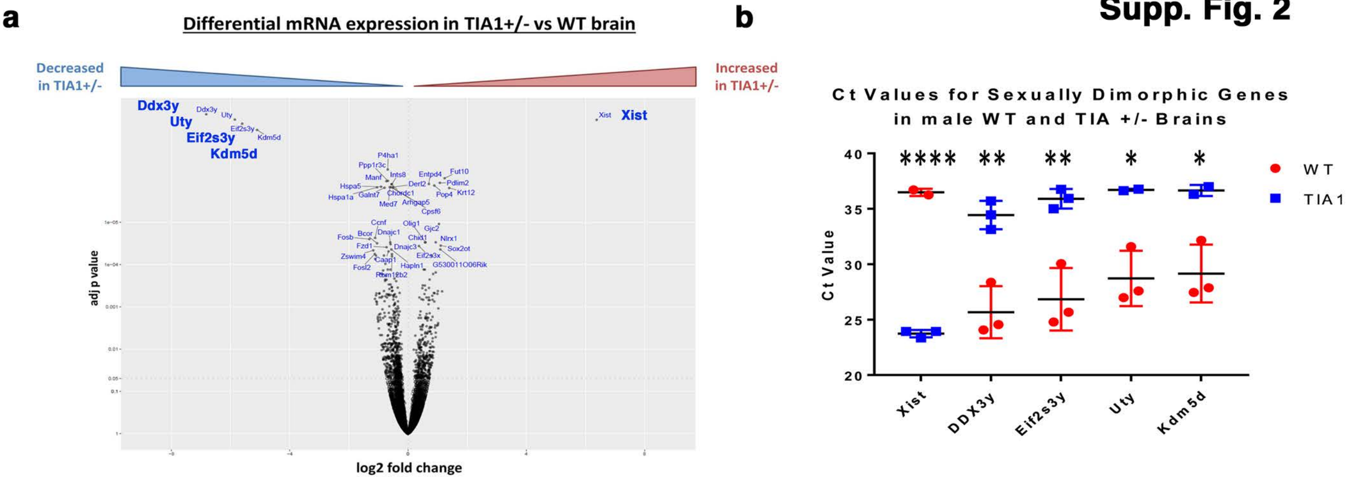
a
Differential mRNA expression in TIA1+/- vs WT brain
Decreased in TIA1+/-
Increased in TIA1+/-
Ddx3y
Uty
Eif2s3y
Kdm5d
Xist
adj p value
log2 fold change
b
Ct Values for Sexually Dimorphic Genes in male WT and TIA +/- Brains
Ct Value
****
**
**
*
*
WT
TIA1
Xist
DDX3y
Eif2s3y
Uty
Kdm5d

**Supplementary Figure 2 (related to Figure 2): TIA1 heterozygosity regulates the expression of sexually dimorphic genes. a.** Volcano plot of average mRNA transcript levels detected in the RNA-seq of 9-month *Tia1*+/- compared to WT cortex. Adjusted p values (y axis) were plotted against the log2-transformed fold change (x axis) in the average level of mRNA transcript in *Tia1*+/- and WT cortex (n=3/group). Note that mRNA transcript levels of sexually dimorphic genes are highly differentially expressed in *Tia1*+/- cortex. **b.** Validation of change in expression levels for Xist, Ddx3y, and Eif2s3y in separate 6-month *Tia1*+/- and WT brain tissue (n=3 males per genotype). DDX3y, p=0.0019; eIF2s3y, p=0.0149; Xist, p=0.0093.

# Supp. Fig. 3

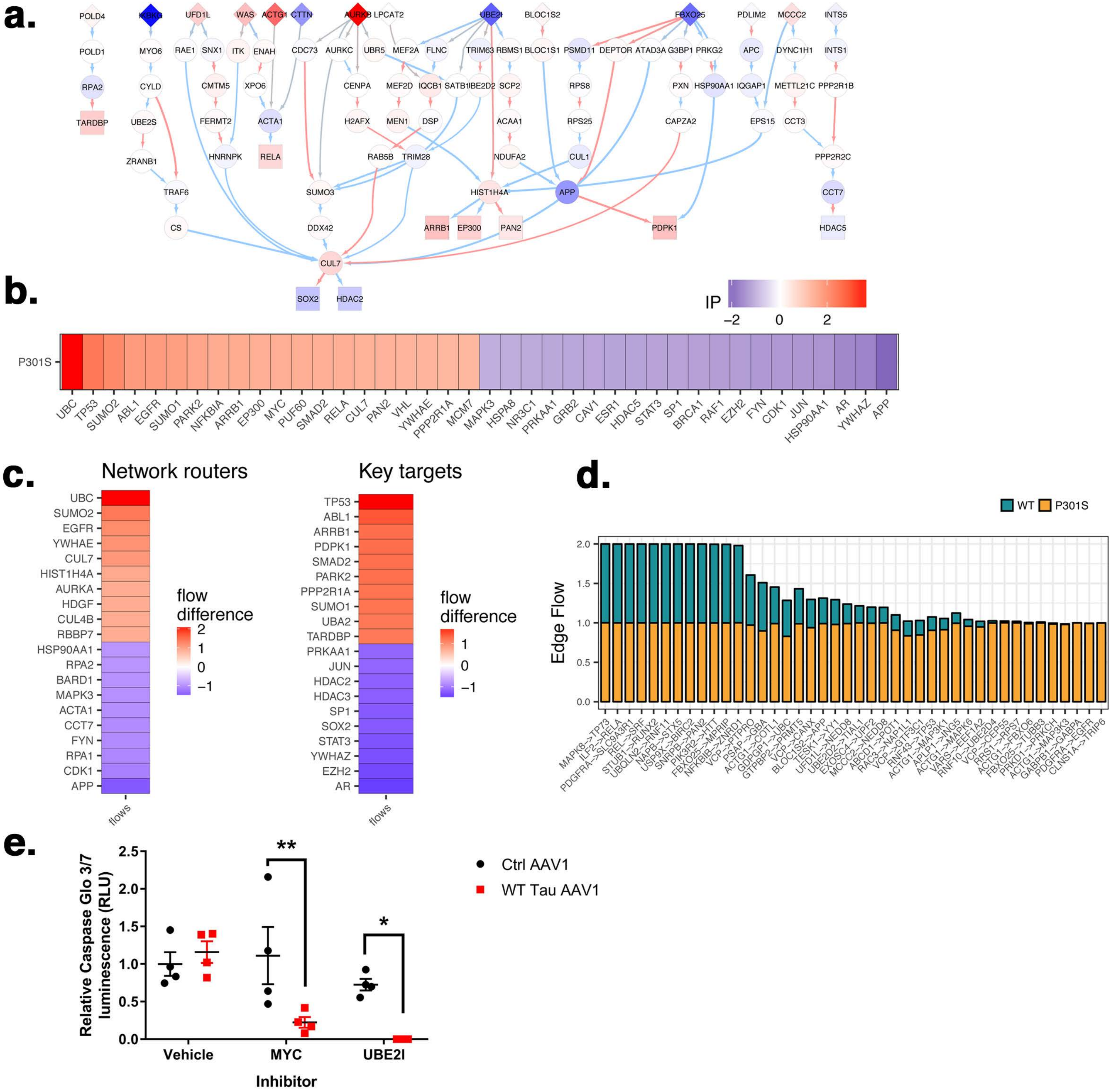
a.
POLD4
IKBKG
UFD1L
WAS
ACTG1
CTTN
AURKB
LPCAT2
UBE2I
BLOC1S2
FBXO25
PDLIM2
MCCC2
INTS5
POLD1
MYO6
RAE1
SNX1
ITK
ENAH
CDC73
AURKC
UBR5
MEF2A
FLNC
TRIM63
RBMS1
BLOC1S1
PSMD11
DEPTOR
ATAD3A
G3BP1
PRKG2
APC
DYNC1H1
INTS1
RPA2
CYLD
CMTM5
XPO6
CENPA
MEF2D
IQCB1
SATB1
UBE2D2
SCP2
RPS8
PXN
HSP90AA1
IQGAP1
METTL21C
PPP2R1B
TARDBP
UBE2S
FERMT2
ACTA1
H2AFX
MEN1
DSP
ACAA1
RPS25
CAPZA2
EPS15
CCT3
ZRANB1
HNRNPK
RELA
RAB5B
TRIM28
NDUFA2
CUL1
PPP2R2C
TRAF6
SUMO3
HIST1H4A
APP
CCT7
CS
DDX42
ARRB1
EP300
PAN2
PDPK1
HDAC5
CUL7
SOX2
HDAC2
b.
IP
−2
0
2
P301S
UBC
TP53
SUMO2
ABL1
EGFR
SUMO1
PARK2
NFKBIA
ARRB1
EP300
MYC
PUF60
SMAD2
RELA
CUL7
PAN2
VHL
YWHAE
PPP2R1A
MCM7
MAPK3
HSPA8
NR3C1
PRKAA1
GRB2
CAV1
ESR1
HDAC5
STAT3
SP1
BRCA1
RAF1
EZH2
FYN
CDK1
JUN
HSP90AA1
AR
YWHAZ
APP
c.
Network routers
UBC
SUMO2
EGFR
YWHAE
CUL7
HIST1H4A
AURKA
HDGF
CUL4B
RBBP7
HSP90AA1
RPA2
BARD1
MAPK3
ACTA1
CCT7
FYN
RPA1
CDK1
APP
flows
flow difference
2
1
0
−1
Key targets
TP53
ABL1
ARRB1
PDPK1
SMAD2
PARK2
PPP2R1A
SUMO1
UBA2
TARDBP
PRKAA1
JUN
HDAC2
HDAC3
SP1
SOX2
STAT3
YWHAZ
EZH2
AR
flows
flow difference
1
0
−1
d.
WT
P301S
Edge Flow
2.0
1.5
1.0
0.5
0.0
MAPK8->TP73
ILF2->RELA
PDGFRA->SLC9A3R1
REL->SRF
STUB1->RUNX2
UBQLN2->RNF11
NAPB->STX5
USP9X->BIRC2
SNRPB->PAN2
PIK3R2->HTT
FBXO25->MPRIP
NFKBIB->ZNRD1
VCP->PTPRO
PSAP->GBA
ACTG1->COTL1
GDPGP1->UBC
GTPBP2->PRMT5
VCP->CANX
BLOC1S2->APP
TESK1->YY1
UFD1L->NEDD8
UBE2D2->TIAL1
EXOSC4->UPF2
MCCC2->NEDD8
ABCD1->POT1
RAC3->NAP1L1
VCP->GTF3C1
RNF43->TP53
ACTG1->MAP3K1
APLP1->ING5
ACTG1->MAPK6
VARS->EEF1A2
RNF10->UBE2D4
VCP->CEP55
RRS1->RPS7
ACTG1->FBXO6
FBXO25->TUBB3
PRKD1->PRKCH
ACTG1->MAP3K3
GABPB1->GABPA
PDGFRA->EGFR
CLNS1A->TRIP6
e.
Relative Caspase Glo 3/7 luminescence (RLU)
2.5
2.0
1.5
1.0
0.5
0.0
**
*
Ctrl AAV1
WT Tau AAV1
Vehicle
MYC
UBE2I
Inhibitor

**Supplementary Figure 3 (related to Figures 5 and 6): Disease phenotype in PS19 vs WT mice is regulated by a prioritized biological network.** NetDecoder analysis was performed on the RNA-seq data from 9-month PS19 (P301S *Tia1*+/+) and WT cortex in order to identify context-dependent changes inherent to the disease phenotype in PS19 tauopathy mice, as described previously (Lummertz da Rocha et al., 2016). **A.** Prioritized network consisting of biological pathways predicted to regulate the differences in phenotype between PS19 and WT mice. Red and blue nodes denote proteins exhibiting either increased (red) or decreased (blue) information flow in PS19 compared to WT cortex. Arrows denote direction of information flow from source genes to target genes based on known protein-protein interaction (PPI) data. Network routers (diamonds) are upstream of intermediary (circles) and target (square) protein nodes. **B-D.** Key genes (B), network routers (C), and key targets (D) identified by NetDecoder analysis to mediate context-dependent disease phenotype in PS19 compared to WT cortex. **E.** Inhibition of MYC and UBE2I protects against tau toxicity. Primary hippocampal neurons from neonatal C57BL/6J mice were cultured for 21 days *in vitro* (DIV), and analyzed for caspase activation following 48 h treatment with vehicle control (Veh, DMSO) or a chemical inhibitor of MYC or UBE2I, using the Casp 3/7 Glo kit (Promega). The neurons were previously transduced on DIV 2 with AAVs (serotype 1, AAV1) expressing human WT Tau, human P301L Tau, or vector control (Ctrl). #$p<0.1090$ *$p=0.0435$ **$p=0.0047$ by 2-Way between-subjects ANOVA with Tukey's post-hoc comparisons. Error bars denote means ± SEM (n=4/group).
